# Sex-biased dispersal promotes adaptive parental effects

**DOI:** 10.1186/1471-2148-10-217

**Published:** 2010-07-16

**Authors:** Emmanuelle Revardel, Alain Franc, Rémy J Petit

**Affiliations:** 1 INRA, UMR 1202 Biodiversity Genes & Communities, F-33610 Cestas, France; 2 University of Bordeaux, UMR1202 Biodiversity Genes & Communities, Bordeaux, F-33400 Talence, France; 3 University of Bordeaux, UMR1202 Biodiversity Genes & Communities, F-33610 Cestas, France

## Abstract

**Background:**

In heterogeneous environments, sex-biased dispersal could lead to environmental adaptive parental effects, with offspring selected to perform in the same way as the parent dispersing least, because this parent is more likely to be locally adapted. We investigate this hypothesis by simulating varying levels of sex-biased dispersal in a patchy environment. The relative advantage of a strategy involving pure maternal (or paternal) inheritance is then compared with a strategy involving classical biparental inheritance in plants and in animals.

**Results:**

We find that the advantage of the uniparental strategy over the biparental strategy is maximal when dispersal is more strongly sex-biased and when dispersal distances of the least mobile sex are much lower than the size of the environmental patches. In plants, only maternal effects can be selected for, in contrast to animals where the evolution of either paternal or maternal effects can be favoured. Moreover, the conditions for environmental adaptive maternal effects to be selected for are more easily fulfilled in plants than in animals.

**Conclusions:**

The study suggests that sex-biased dispersal can help predict the direction and magnitude of environmental adaptive parental effects. However, this depends on the scale of dispersal relative to that of the environment and on the existence of appropriate mechanisms of transmission of environmentally induced traits.

## Background

Whether sessile or not, all organisms experience environmental heterogeneity. As a consequence, divergent selection takes place, leading to local adaptation, unless selection is opposed by the homogenising effects of gene flow [1,2]. Local adaptation is defined by the difference in performance between conspecific individuals of local and non-local origins. If dispersal is sex-biased, intermediate situations arise with individuals having the parent of one sex (male or female) of local origin but not the other. This could create suitable conditions for the evolution of environmental adaptive parental effects, with offspring selected to perform like their nearest, locally adapted parent. Hence, sex-biased dispersal could help predict the direction and magnitude of adaptive parental effects.

In a sexually reproducing species, parental effects occur when the phenotype of an individual is determined more strongly by one of its parents, beyond the equal contribution expected from biparental inheritance [3-5]. Parental effects are ubiquitous in nature and have been detected at a wide range of traits both in animals and in plants [5-10]. Parental effects can be due to genetic, epigenetic, behavioural or cultural inheritance. The corresponding mechanisms are extremely varied and include cytoplasmic inheritance, segregation distortion, parental imprinting, transgenerational plasticity (as the result of the transmission of information derived from parental quality or parental environment), parental care, or learning ability [3,11-14]. As a consequence, research on parental effects is considered to be at the forefront of the ongoing integration of development, ecology and evolution [15-17].

Not all parental effects increase offspring fitness. In vertebrates, the adverse effects on the fitness of offspring of thousands of substances ingested by parents, such as drugs, food additives, pesticides, metals, has been well established [8]. Other parental effects can have positive effects on offspring fitness, but regardless of the environment. However, a growing number of parental effects have been described that contribute to offspring adaptation to local abiotic or biotic environmental conditions [18-21]. For instance, in an understorey herb, *Campanula americana*, maternal light environment affects offspring life history in ways that should increase their fitness under similar light conditions [22,23]. In *Sorghum bicolour*, salt-treated mother plants produce a higher proportion of salt-adapted offspring [24]. In spruce, timing of budset is regulated by a memory of temperature during zygotic embryogenesis, i.e., when the seed is still attached to the mother, resulting in trees that are better adapted to the climate where the mother trees are growing [25,26]. In amphibians, local adaptation to water acidity is mediated by maternal effects [27]. Examples involving biotic factors have also been studied, especially cases of induced resistance. For instance, wild radish plants damaged by herbivores during growth have been shown to induce resistance of the plants' progeny compared to controls, potentially contributing to local adaptation in infested areas [28]. Similarly, in yellow monkeyflower, herbivory damage on early leaves induces increased production of glandular trichomes on later leaves but also in the maternal progeny before they experience herbivory, a plastic response that is likely adaptive [29]. In insects, there are many examples of adaptive transgenerational responses to predators [30] or to plant host quality [e.g. [31]]. In vertebrates, one of the best-characterized cases of adaptive maternal effects is transgenerational inheritance of mothering style in rats. Adoptive offspring of mothers with high or low levels of grooming and nursing have predictable differences in DNA methylation at a glucocorticoid receptor gene promoter in the hippocampus [32]. These epigenetic changes result in differential sensitivity to adversity and increase the probability of offspring survival to sexual maturity in the corresponding environments [33]. Similarly, in humans, the increased levels of insulin resistance in offspring of mothers starved during pregnancy has been hypothesised to provide adaptation later in life in environments where nutrition is poor, at the expense of increased diseases risks, an interpretation that is however still debated [16].

Hence, environmental adaptive parental effects are the focus of increasing attention. Yet, the origin of adaptive parental effects remains poorly understood. Parental effects are often considered to be physiological inevitabilities [21] or to represent a form of transgenerational developmental noise [16]. For instance, anisogamy and internal fertilization typically lead to greater maternal than paternal effects. However, such explanations do not account for the evolution of environmental adaptive parental effects.

Recently, Galloway [22] and Galloway and Etterson [23] proposed that environmental adaptive parental effects could have evolved as a source of adaptive plasticity between generations. They argued that sex-biased gene dispersal in plants should select for environmental adaptive maternal effects in heterogeneous environments. This is because young seedlings should experience an environment more similar to that of their mother than to that of their father, because in plants seed dispersal is typically reduced compared to pollen dispersal. If local environments were predictable across generations, environmental maternal effects could provide a mean for maternal plants to adjust the phenotype of their offspring and enhance its success in the environment that it is likely to encounter. A similar idea was formulated by Spencer and Clark [34] for genomic imprinting in mammals. These authors suggested that genomic imprinting, a case of parental epigenetic effect [35], could have evolved as a consequence of selection to become similar to the mother. In mammals, dispersal is often male-biased [36,37]. If females are locally adapted, but not newly immigrated males, it would be advantageous for offspring of both sexes to resemble their mother more so than their father [34].

Although the studies of Galloway [22] and Spencer and Clark [34] outline a potentially general mechanism for the origin and evolution of environmental adaptive parental effects, they discuss it in a limited context (seed plants in one case, mammals in the other) and do not quantify the impact of asymmetric dispersal and habitat structure on the intensity of selection for parental effects. Here we present a simple model with a static patchy environment and variable ratios of male to female dispersal rates and use it to estimate the probability with which an offspring will reside in the same habitat as its mother or father. This allows us to make predictions as to when selection should favour the emergence of adaptive parental effects. We contrast the situation for plants, whose dispersal is intrinsically asymmetric because they disperse their genes through haploid pollen and diploid seeds, with that of other organisms where dispersal is not constrained in the same way. We then outline a strategy to test the predictions of the model by confronting them with findings from empirical studies and discuss model limitations and possible directions for improvement.

## Results

**Figure 1:**
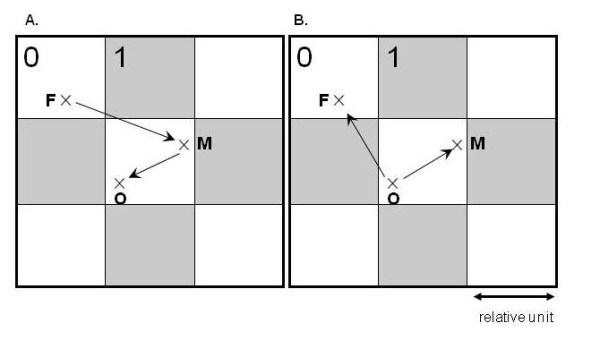
**Simulation of dispersal on a checker board**. A. The plant model. B. The animal model. The white (0) and grey (1) squares represent environmental heterogeneity. Position of each individual is shown by a cross: F for father, M for mother, O for offspring. Arrows represent how dispersal was modelled. Dispersal is relative to the scale of the environment.

Simulations were used to estimate the probabilities for the offspring to reside in the same environment than each of its parents, assuming two contrasted environments distributed regularly, as on a checkerboard (Figure 1). These probabilities depend on pollen and seed dispersal, in plants, and on male and female dispersal, in animals, which were varied relative to the scale of the environmental patches. A simple exponential kernel was used to model each dispersal curve, of parameter *β*. We then compared the advantage of the strategy of maternal or paternal transmission of fitness (*Z*_*m *_or *Z*_p_) relative to the strategy of biparental inheritance of fitness, in the case of maximum contrast between environments, as detailed in the Methods section.

### Plant model

**Figure 2:**
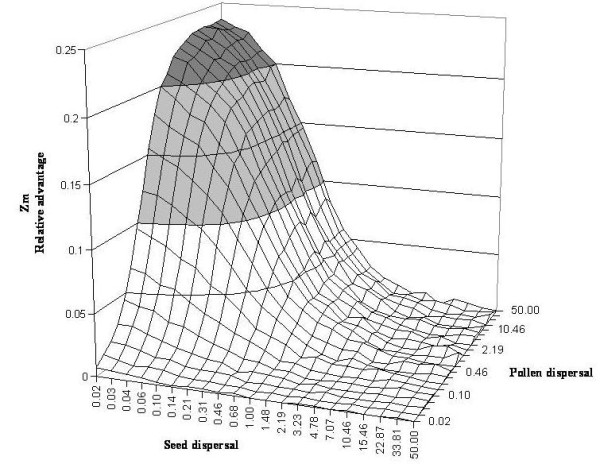
**The relative advantage of the maternal strategy over the biparental strategy, *Z*_m_, for different pollen and seed dispersal parameters in the plant model**. Dispersal parameters are relative to the scale of the environment.

In the plant model, when mean seed dispersal is close to or lower than the size of environmental patches, there is an advantage for a maternal mode of inheritance compared to biparental inheritance (*Z*_m _> 0, Figure 2). This is especially true if pollen dispersal is high, but it holds even when pollen dispersal is lower than seed dispersal. If mean seed dispersal is much larger than the size of environmental patches, there is no more advantage for the maternal strategy (*Z*_m _~ 0). There is no parameter space where paternal inheritance is favoured by selection (i.e., where *Z*_m _< 0), even when pollen dispersal is much lower than seed dispersal. *Z*_m _is maximal (values close to 0.25) when seed dispersal is close to zero (*β*_s _= 0.02) and pollen dispersal close to the maximum (*β*_p _= 50), which corresponds to the maximal probability for an offspring to be in the same environment than its mother but in a different environment from its father.

### Animal model

**Figure 3:**
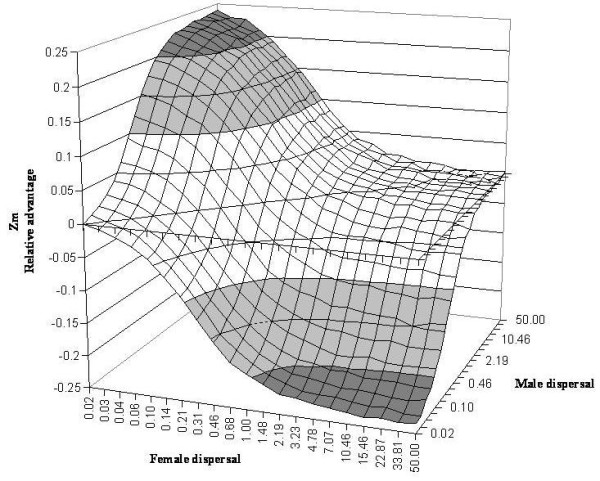
**The relative advantage of the maternal strategy over the biparental strategy, *Z*_m_, for different male and female dispersal parameters in the animal model**. Dispersal parameters are given relatively to the scale of the environment.

On the contrary, in the animal model, the situation is symmetric, with an advantage for maternal inheritance (0 ≤ *Z*_m _≤ 0.25) when females disperse less than males and an advantage for paternal inheritance (-0.25 ≤ *Z*_m _≤ 0, i.e. 0 ≤ *Z*_p _≤ 0.25) when males disperse less than females (Figure 3). *Z*_m _is maximal (values close to 0.25) when female dispersal is close to zero (*β*_*♀ *_= 0.02) and male dispersal close to the maximum (*β*_*♂ *_= 50), which corresponds to the maximum probability for an offspring to be in the same environment than its mother but in a different environment from its father. Similarly, *Z*_m _is minimal (values close to -0.25) when female dispersal is maximum (*β*_*♀ *_= 50) and male dispersal close to zero (*β*_*♂ *_= 0.02), which corresponds to the maximal probability for an offspring to be in the same environment than its father but in a different environment from its mother.

### Models comparison

**Figure 4:**
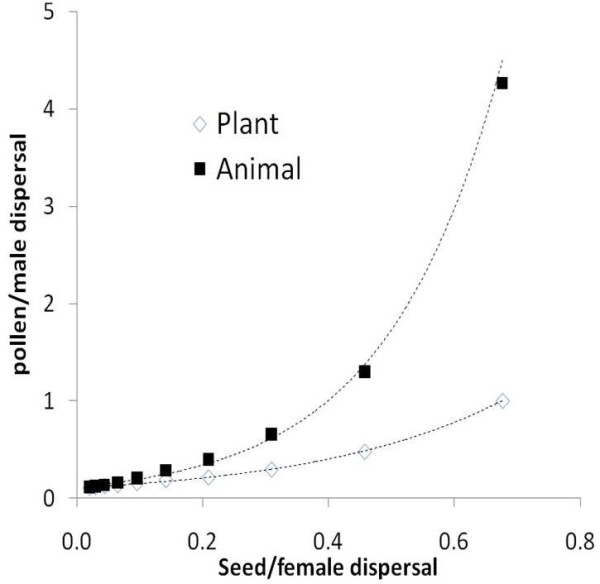
**Minimum pollen or male dispersal values needed for *Z*_m _to reach 0.05, as a function of seed or female dispersal**. For similar seed or female dispersal, much higher male than pollen dispersal values are needed to exert the same selective pressure in favor of maternal effects, illustrating the high propensity of plants to evolve maternal effects.

A closer comparison between results for the plant and the animal models indicates that a higher male/female than pollen/seed dispersal ratio is needed to achieve the same selective pressure in favour of the maternal strategy. For instance, for the maternal strategy to outperform the biparental strategy by 5%, male dispersal in animals has to be about four times as large as pollen dispersal for the same values of female and seed dispersal (for *Z*_m _≥ 0.05 with *β*_*♀ *_= *β*_s _= 0.68, it takes *β*_*♂ *_≥ 4.78 but only *β*_p _≥ 1) (Figure 4).

## Discussion

Our goal was to clarify when environmental adaptive parental effects (whether maternal or paternal) are most likely to evolve as a consequence of sex-biased dispersal and of the spatial heterogeneity of the environment, to test predictions of previous verbal models. We identify parameter space where either maternal or paternal environmental effects are selected for. Two major points emerge. First, the selective pressure to develop environmental adaptive parental effects is particularly high when dispersal is strongly sex-biased, as suggested previously for plants [22]. This selective pressure depends in a complex, non-linear way on the dispersal of seeds and pollen (or males and females) relative to the scale of the environment. Second, the results show that in heterogeneous static environments, plants are not expected to evolve environmental adaptive paternal effects: genes that are transmitted from the male gamete will be dispersed not only by pollen but also by seeds, so, on average, they will be dispersed over larger distances than genes inherited maternally, which are dispersed only by seeds. In contrast, animals are expected to evolve either environmental adaptive maternal or paternal effects, depending on whether dispersal is male-biased or female-biased. Another related difference is that, for adaptive maternal effects to evolve in animals, male dispersal needs to be higher than female dispersal. Instead, in plants, adaptive maternal effects can evolve even if pollen dispersal is lower than seed dispersal.

### Model limitations

The model used made a number of assumptions that should be borne in mind. Below, we outline some of these limitations and discuss whether they could affect its performance.

First, we considered only two contrasted environments in the model. As a consequence, an offspring dispersing far away from its parents still has a 50% chance to be located in an environment identical to its natal one. If there were a larger set of environmental conditions, then a larger fraction of dispersing offspring would encounter a new environment (up to 100% if there are as many environments as patches on the landscape). The strategy whereby the fitness of the offspring is controlled by the environment of the least dispersing sex could then in theory outperform the biparental strategy by up to 50%, compared to only 25% in the current model, when assuming maximum contrast between the fitness of individuals in the two environments. Instead, for less extreme contrasts between environments, the selective pressure in favour of uniparental inheritance is decreased proportionally.

Second, we considered a fixed environment. Previous studies have shown that when environmental change is rapid or cyclical, adaptive plasticity can evolve, '*by eliminating the lag-time associated with de novo induction of the phenotype in offspring*' [38]. In the case of adaptation to environmental change, the rapidity of the response is critical [16]. Hence, it would be interesting to relax the assumption of static environment in our model and test how this affects predictions for the evolution of parental effects.

Finally, we did not consider physiological, developmental and genetic constraints [e.g. [39,40]. The model only predicts, under relatively restrictive conditions (including a fixed dispersal strategy), the *potential *for environmental adaptive parental effects to evolve. In practice, environmental parental effects (which imply some form of memory) will evolve only if the mechanisms exist to store and transfer the relevant information over generations. The parental environment can affect the offspring fitness at two stages: before and after fertilisation (i.e. prezygotic and postzygotic effects). Postzygotic environmental parental effects are more direct because they involve the developing offspring itself. They are facilitated by the development of viviparity (e.g. in plants and in mammals) and by parental care in animals and resource provisioning in plants. Prezygotic parental effects are necessarily more indirect. Moreover, earlier work has shown that the evolution of parental effects can be limited by genetic constraints such as recombination rates [34]. These constraints need to be kept in mind when attempting to check the predictions of the model.

### Prospects to test the predictions of the model using empirical evidence

Parental effects have been described in a large number of organisms, but comparable data are rare [but see [14]]. Moreover, our model applies to environmental adaptive parental effects, not to parental effects that are maladaptive, neutral or that provide general rather than local selective advantage [21]. As many studies measure parental effects without checking whether these are adaptive, comparisons might not always be meaningful. Moreover, because the relevant geographic scale of the environment depends on the trait considered (e.g. growth, drought tolerance, disease tolerance, etc.), the selective pressure to develop parental effects should vary depending on the trait [22]. Only data pertaining to the same trait in the same environment are strictly comparable. Notwithstanding these difficulties, we outline below some prospects to test the theory in plants and in animals.

#### Plants

In plants, at the rangewide scale, historical levels of pollen flow have been estimated to be at least an order of magnitude larger than levels of seed flow [median of the pollen-to-seed migration ratio = 17, ref. [41]]. The strong asymmetry of pollen and seed dispersal distances, combined with the intrinsically biased dispersal system of plants (only male gametophytes disperse) and the sessile habit, suggest that environmental adaptive maternal effects should be large and paternal effects virtually absent in plants. Maternal effects have indeed been frequently reported in plants, whereas paternal effects have been considered to be negligible [e.g. [5]]. In the few studies were environmental paternal effects were detected, they were of limited magnitude and often equivocal [42], or were dependent on an interaction with the maternal plant [43]. Hence, the results are compatible with the expectation for an absence of environmental paternal effects in plants. However, the comparison appears unbalanced because there are many physiological and developmental pathways by which maternal effects can arise in plants [e.g. [44]], but few that could allow the evolution of any kind of paternal effect. In fact, unlike in animals, the only way whereby the plant paternal environment could influence the offspring is prezygotic [45]. Hence, the mechanisms by which paternal environments could influence the offspring phenotype are necessarily indirect and limited [3,46]. To better evaluate the model's predictions, comparative studies should instead investigate if variation in the relative dispersal of pollen and seed across plant species is associated with a corresponding variation in the intensity of maternal effects. Interestingly, pollen/seed dispersal ratios are not universally large in plants. In particular, in autogamous (i.e. predominantly selfing) plants, pollen dispersal is lacking or is very limited [e.g., [47]], to the point that gene flow should no longer be sex-biased. One could therefore predict that outcrossed offspring of predominantly autogamous plants should display less (locally adaptive) maternal effects than offspring of closely related allogamous plants.

#### Animals

In most animals with separate sexes, dispersal of genes originating from male and female gametes is not constrained as it is in plants. Hence, a greater diversity of adaptive parental effects is expected, depending on which sex is the main disperser. In particular, the model predicts the evolution of adaptive paternal effects when males are more philopatric than females (as well as the opposite, i.e. the evolution of adaptive maternal effects when females are more philopatric than males). Nevertheless, the mechanisms that could allow the expression of paternal effects in animals, although not as unlikely as in plants, are less numerous than those favouring the expression of maternal effects. In particular, the only universal difference between the two sexes, the size difference between sperm and egg cells [48], already represents a significant prezygotic obstacle for the development of paternal effects. By contrast, postzygotic mechanisms are not so constrained. In particular, paternal care, which could lead to the development of at least some paternal effects [14], has evolved repeatedly in animals, including in polychaetes, hemipters, amphibians and birds, and at high frequency in sea spiders and fish [49,50]. Previous reviews indicate that paternal care is associated with site-attached behaviour by males [e.g. [49,50]]. Hence, given that site-attached behaviour by one sex should typically imply stronger philopatry for that sex, the prediction that adaptive parental effects will be biased towards the more philopatric sex is not without substance, at least for traits that can be influenced by parental behaviour [14]. However, this reasoning merely suggests that the model is plausible and worthy of further investigation.

In mammals, dispersal is often male-biased [36,37], so maternal effects should predominate, according to our model. In the only review available on the direction and intensity of parental effects in animals (using literature data on reciprocal crosses), mammals were indeed shown to be characterized by strong maternal effects for a number of traits [14]. These findings are therefore compatible with our model, but whether sex-biased dispersal is the cause of this trend cannot be easily assessed, because of the numerous physiological and developmental pathways that facilitate maternal effects in mammals. Humans are unusual among mammal, as about 70% of human societies practice some form of patrilocality [e.g. [51]], especially since the emergence of agriculture [52]. So in humans, unlike in most mammals, adaptive paternal effects are expected, provided that suitable mechanisms exist that allow their expression. An interesting case is that of surnames, which are inherited from the father in many human societies [53]. Surnames are not without consequence and can be locally adaptive [e.g. [54]]. This example illustrates that adaptive parental effects can involve cultural traits and can respond rapidly to changing environments and sex-related dispersal patterns, which could be useful to test our model.

In birds, there is a general pattern of female-biased dispersal [37], although several species show no sex-biased dispersal and a few have male-biased dispersal [55]. Hence one would predict that adaptive paternal effects should be more frequent in birds than in mammals. There is a tendency for maternal effects to be weaker in birds than in mammals [14]. However, most reports of parental effects in birds still describe maternal, not paternal effects [56]. It seems therefore that anatomical, developmental and other constraints can be more important than sex-biased dispersal in helping predict the direction of parental effects. Note however that our model only predicts the direction of locally adaptive parental effects, not of all parental effects. While prezygotic paternal effects might be rare in birds, postzygotic paternal effects could be more frequent. An example of such locally adaptive paternally transmitted feature in birds is males' song, a learned behavior [e.g. [57,58]]. There is strong evidence that local courtship song structure in male house finches is associated with locally adaptive modifications of bill form, function, and development [59].

## Conclusions

The above examples suggest that the model's predictions, although somewhat limited by the importance of developmental constraints, are testable. The comparisons could focus on closely related species (or populations of the same species). Ideally, a full analysis would imply systematic quantification of sex-biased dispersal and of parental effects for a few well-chosen traits and the use of phylogenetically-based comparative approaches.

During the last years, a few studies have started to explore the evolutionary consequences of sex-biased dispersal. For instance, Johnstone and Cant [60] and Gardner [61] have shown how sex-biased dispersal of adults mediates the evolution of altruism. Guillon et al. [62] showed that the combination of sex-biased dispersal of gametes and variation of habitat quality modifies sex allocation in animals as well as in plants, whereas Lopez *et al*. [63] found that sex-biased dispersal in plants can affect migration load. Our study confirms that sex-biased dispersal has potentially profound evolutionary consequences that deserve further investigations. It also contributes to the growing awareness that other routes than pure biparental genetic inheritance can result in adaptation to local environments [16,64].

## Methods

### Environment

The diversity of environments is often summarized in binary form (wet versus dry, calcareous versus acidic, etc.). We therefore used a simple static and regular pattern with square cells as on a checkerboard, with two alternating states, one for each environment, while acknowledging that this is a rough simplification. The size of each cell is arbitrarily set to one and is constant across simulations. On the contrary, dispersal distances (see below) were varied over several orders of magnitude above and below cell size. Hence, dispersal distances are expressed in relative scaling units compared to the environment.

### Dispersal function

Several dispersal kernel functions have been used to model dispersal [65,66]. Some of the most frequently used are the exponential, Gaussian, or power law functions. They differ in particular by the way the tail distribution is modelled [67]. For this first-order exploratory approach, we have selected a simple kernel (exponential function: *f*(*x*) = *βe*^-*βx*^) for dispersal distances. This model of dispersal has only one degree of freedom (*β*), allowing to more easily explore the variability of the response. However, we also made the calculations for the two other classical dispersal kernels (Gaussian and power law), and the findings were very similar to those obtained with the exponential function (results not shown). In all cases, a uniform random variable was used to select the angle of dispersal. For such a work, we need to associate two dispersal curves (i.e. pollen and seed, for the plant model), which is implemented by a convolution of both dispersal curves. This is tractable in one dimension, but we could not find an analytical solution in a plane (two dimensions). Therefore, our modelling work relied on a Monte Carlo method, based on numerical simulations with a high number of repetitions.

### The plant model

Pollen and seed dispersal are considered to take place on a two dimensional infinite plane. The point at the origin of the x and y axes represents the location of the father (since we are interested in relative distances, the process is invariant through translations, and actual locations do not matter). The dispersal of the pollen is then simulated using the pollen dispersal function. The arrival point represents the location of the mother. The dispersal of the seed is then simulated using the seed dispersal function, starting from the mother's location. The arrival point represents the location of the offspring (Figure 1a). The "plant model" of dispersal is intrinsically asymmetric because a gene inherited through the male gamete will be dispersed through the pollen and then through the seed, whereas a gene inherited through the female gamete will only be dispersed through the seed. As a consequence, an offspring will (on average) be closer to its mother than to its father.

### The animal model

In contrast, in animals, dispersal of genes inherited through the male gamete can be more restricted than that of genes inherited through the female gamete, if males are more philopatric than females, so that an offspring could (on average) be born nearer to the place of origin of its father than to that of its mother. We therefore simulated another model in which the distance between the offspring and its father was not constrained by the position of the mother. In this case, the point at the origin of the x and y axes represents the location of the offspring and the position of the father and of the mother are determined using a dispersal function for the males and the females (Figure 1b). The distances thus simulated correspond to the distance between the mother's and father's place of birth and the offspring's place of birth, as when dispersal of animals is restricted to the movement of juveniles from birth place to site of first reproduction (natal dispersal).

### Running the simulations

Each dispersal parameter *β *was varied from 0.02 to 50 (i.e. up to 50 times smaller and 50 times larger than the characteristic scale of the environment), using a homogenous logarithmic increase, with 20 steps (i.e. the ratio between two consecutive values is constant). The total number of combinations of pollen and seed dispersal parameters (*β*_p _and *β*_s_) and of male and female dispersal parameters (*β*_♂ _and *β*_♀_) amounts to 441 cases (21 × 21). For each pair of dispersal parameters, 20,000 runs were implemented (total of 8.82 millions runs for each of the two models). In all runs, the state of the cells occupied by the father, the mother and the offspring were compiled. Sites are labelled 0 and 1 as for any binary state. In the plant model, assuming that the father always lives in the same site does not lead to a loss of generality. Similarly, in the animal model, assuming that the offspring always live in the same site does not lead to a loss of generality. For instance, in the plant model, the father is always in state 0. There are then four possibilities for the sites inhabited by the father, the mother and the offspring, respectively (0,0,0), (0,0,1), (0,1,0) and (0,1,1). We have computed the numbers (*n*_000_, *n*_001_, *n*_010_, *n*_011_, called *n*_1_, *n*_2_, *n*_3_, *n*_4_), corresponding to each of the four cases, with *n*_1 _+ *n*_2 _+ *n*_3 _+ *n*_4 _= 20,000. In the animal model, the sites inhabited by the father, the mother and the offspring are instead: (0,0,0), (0,1,0), (1,0,0) and (1,1,0), otherwise the procedure is the same (Table 1).

**Table 1:** Derivation of the mean adaptive value of offspring for each of the three strategies of inheritance (biparental, maternal and paternal)

Environnement^1^						Offspring fitness		
**Father**	**Mother**	**Offspring**	**Case *i***	**Number of cases *i***	**Probability of case *i***	**biparental**	**maternal**	**paternal**

0/0	0/0	0/0	1	n1	p1	1	1	1
0/1	0/1	1/0	2	n2	p2	α	α	α
0/0	1/1	0/0	3	n3	p3	(1 + α)/2	α	1
0/1	1/0	1/0	4	n4	p4	(1 + α)/2	1	α

Total				20,000	1	w_b _= p1 + αp2 + (1+ α)(p3 + p4)/2	w_m _= p1 + p4 + α(p2 + p3)	w_p _= p1 + p3 + α(p2+ p4)

^1 ^Values before the slash correspond to the plant model and after the slash to the animal model. In the plant model, the father is always in state 0, in the animal model, the offspring is always in state 0

### Measuring the advantage of uniparental versus biparental transmission of adaptive modifications

To evaluate the relative advantage of different mechanisms of inheritance of fitness-related characters, we consider three extreme strategies: maternal, paternal and biparental inheritance. The "maternal" strategy corresponds to a scenario where the fitness of the offspring is entirely determined by the mother's environment. If the mother's environment is similar to that of the offspring, then the fitness of the offspring is maximal (value of one). If not, its fitness is given a value of α (with 0 ≤ α < 1). Symmetrically, in the "paternal" strategy, the offspring fitness depends only on the correspondence of the offspring environment with that of its father. In the "biparental" strategy, the offspring fitness is determined equally by the environment of the two parents. It is equal to one if both parents are located in the same environment as the offspring, (1+ α)/2 if only one of the two parents is located in the same environment than the offspring, and α if none of the parents are located in the same environment than the offspring. Predicted offspring fitness under each scenario is displayed in Table 1 for the plant and animal models. The probability of each of the four possible configurations of environments for mother, father, and offspring (obtained through simulations) is multiplied by the corresponding fitness of the offspring under each strategy of transmission of fitness. The average adaptive value of each strategy (w_m_, w_p _and w_b_) is obtained by computing the sum of the four products (probability of each configuration multiplied by its corresponding fitness, as indicated in Table 1). The relative advantage of the maternal strategy over the biparental strategy, noted *Z*_m_, is:

$$\begin{array}{l} Z_{\text{m}}={\text{w}}_{\text{m}}-{\text{w}}_{\text{b}}\\ =[\text{p}1+\text{p}4+\alpha (\text{p}2+\text{p}3)]-[\text{p}1+\alpha \text{p}2+(1+\alpha )(\text{p}3+\text{p}4)/2]\\ =(1-\alpha )(\text{p}4-\text{p}3)/2 \end{array}$$

Similarly, the relative advantage of the paternal strategy over the biparental strategy, noted *Z*_p_, is:

$$\begin{array}{l} Z_{\text{p}}={\text{w}}_{\text{p}}-{\text{w}}_{\text{b}}\\ =[\text{p}1+\text{p3}+\alpha (\text{p}2+\text{p4})]-[\text{p}1+\alpha \text{p}2+(1+\alpha )(\text{p}3+\text{p}4)/2]\\ =(1-\alpha )(\text{p3}-\text{p4})/2 \end{array}$$

Therefore, as expected, the relative advantage of the maternal strategy (*Z*_m_) is opposite to that of the paternal strategy (*Z*_p_). Moreover, both *Z*_m _and *Z*_p _are strictly proportional to the difference in fitness between the two environments (1 - α).

## Authors' contributions

RJP and ER conceived the study together, ER then elaborated on this proposal. AF wrote the program and performed the simulations. The study then evolved through a close collaboration and numerous discussions between all three authors. RJP and ER drafted the manuscript. All authors read and approved the final version of the manuscript.
